# Hot flushes and sweating, sleep problems, joint and muscular discomfort, and physical and mental exhaustion in breast cancer survivors during the first 24 months of tamoxifen therapy: a prospective observational study

**DOI:** 10.3389/fonc.2022.844926

**Published:** 2022-08-02

**Authors:** Sumi Sung, Yul Ha Min, Seul Ki Park, Sae Byul Lee

**Affiliations:** ^1^ College of Nursing, Seoul National University, Seoul, South Korea; ^2^ College of Nursing, Kangwon National University, Kangwon-do, South Korea; ^3^ Department of Nursing, Daejeon University, Daejeon, South Korea; ^4^ Division of Breast Surgery, Department of Surgery, University of Ulsan College of Medicine, Asan Medical Center, Seoul, South Korea

**Keywords:** adjuvant endocrine therapy, breast cancer, tamoxifen, signs and symptoms, premenopause

## Abstract

**Purpose:**

This study aimed to explore symptom trajectories over 24 months for hot flushes and sweating, sleep problems, joint and muscular discomfort, and physical and mental exhaustion experienced by premenopausal women diagnosed with tamoxifen-treated breast cancer.

**Methods:**

A total of 104 patients participated in the study. The menopausal symptoms were examined using the Menopausal Rating Scale at baseline, 3–6, 12, and 18–24 months after initiating tamoxifen. The changes over four time points were analyzed using repeated measures analysis of variance. The chi-square test was used to examine the differences between “no symptom-to-mild” and “moderate-to-extremely severe” 3–6 months after initiating tamoxifen according to the patients’ chemotherapy treatment experiences.

**Results:**

All menopausal symptoms occurred in > 70% of patients with breast cancer and persisted until 24 months. More than 50% of patients experienced four menopausal symptoms, with at least two at a serious severity level after initiating tamoxifen. Hot flushes and sweating occurred in the highest number of patients, recording high scores. Sleep problems and physical and mental exhaustion exhibited relatively high scores, even before tamoxifen initiation. There were significant changes over four time points in all symptoms. Young patients aged < 40 years experienced more severe sleep problems, and patients who had previously received chemotherapy experienced more severe joint and muscular discomfort.

**Conclusions:**

This study’s findings may assist in alerting healthcare providers to menopausal symptoms that develop during tamoxifen therapy and the need for early and active intervention to minimize symptom occurrence and distress.

## Introduction

Breast cancer (BC) is the most common cancer among Korean women. A total of 23,647 new BC patients were recorded in 2018 in Korea, and the incidence has continuously increased since 1999, with an average annual increase rate of 4.6% (1). Over 75% of these BCs are estrogen receptor positive, and they are amenable to treatment with adjuvant endocrine therapy (AET). There are two types of AET treatment, tamoxifen (TAM) and aromatase inhibitors (AIs). Among them, TAM has historically been prescribed for ≥ 5 years in premenopausal women with estrogen receptor-positive BC, whereas AIs have been prescribed for postmenopausal women. Tamoxifen therapy has resulted in decreased BC recurrence and mortality rates by 39% and 31%, respectively (2–5).

Current guidelines recommend that BC survivors extend adjuvant TAM therapy from 5 to 10 years to prevent recurrence and increase overall survival (3, 5). This increase in treatment duration implies that increasing numbers of women may be suffering from several menopausal symptoms as the most common side effects of TAM (6). Among them, hot flushes and sweating (HFS), sleep problems (SP), joint and muscular discomfort (JMD), and physical and mental exhaustion (PME) were reported to occur in most patients with BC taking TAM (7–10). These symptoms have been found to develop in Korean patients with BC (11). Despite not being life threatening, these symptoms reportedly impact negatively on patients’ quality of life and undermine TAM adherence (9, 12).

One of the key menopausal-symptom treatments, hormone replacement therapy, is contraindicated in BC survivors due to a potentially increased risk of cancer recurrence (10). Thus, non-hormonal strategies or non-pharmacological therapies have been preferred for of menopausal-symptom treatment in patients with BC (13, 14). To identify interventions that ameliorate symptoms and provide better support throughout a patient’s treatment journey, it is important to understand the patients’ menopausal symptoms and symptom trajectories during TAM therapy. Healthcare providers can better predict the timing of their patient’s menopausal symptoms and concentrate on caring for patients at risk of elevated symptom burden.

Various studies have investigated the menopausal symptoms experienced by patients with BC taking AET. However, previous studies focused on identifying AET-related symptoms with the highest occurrence, intensity, and distress, including cramps, hot flashes, fatigue, eye irritation, and heart discomfort (15–17). Moreover, most research focused on AI-related symptoms rather than those associated with TAM (7). Even if the study focused on TAM-related symptoms, most research has been limited to cross-sectional studies (9, 10, 18, 19). Relevant longitudinal or cohort studies are limited; hence, the changes in symptoms over time have not been fully elucidated.

In addition, chemotherapy has been known to produce a temporary or permanent menopausal status in premenopausal women. Young women receiving adjuvant chemotherapy are known to experience premature menopause, resulting in increased and occasionally abrupt onset of menopausal symptoms (20). These symptoms may prompt young women undergoing chemotherapy to seek treatment for the prevention or amelioration of symptoms associated with TAM-therapy initiation. Most Korean women are diagnosed with BC in their early 40s resulting in > 50% of them being in a premenopausal state (11). Therefore, it is necessary to determine whether menopausal symptoms appear or worsen after TAM initiation in Korean premenopausal women with BC who have already received chemotherapy.

Therefore, this study aimed to explore symptom trajectories over 24 months of four menopausal symptoms, including HFS, SP, JMD, and PME, experienced by premenopausal women diagnosed with BC taking TAM and determine the incidence of menopausal symptoms in patients who would have already undergone chemotherapy upon initiating TAM.

## Materials and methods

### Study design

This was a prospective observational study.

### Participants and procedure

Between November 2016 and April 2017, 556 consecutive women with histologically confirmed BC at Asan Medical Center were screened for eligibility upon admission for surgery. Inclusion criteria were as follows: cases with in situ, stage I, II, or III hormone receptor-positive BC; age at diagnosis ≥ 20 years; and definitive surgery followed by AET, irrespective of chemotherapy. Women with distant metastases at diagnosis (stage IV BC); local or regional recurrent tumors; or medical history of psychiatric or neurologic illness were excluded. The detailed sampling and attrition process has been described in a previous study (21). Among 370 eligible patients, 210 consented to participate in this study (137 declined to participate, and 23 could not be contacted). Among the 210 patients, 107 were excluded (90 withdrew their consent during the study, 8 were lost to follow-up, 2 stopped AET, and 7 developed recurrent BC during the first 24 months of AET). During the hospital stay after breast cancer surgery, subjects were contacted by a clinical research nurse. After obtaining consent, patients completed paper-based questionnaires at the first visit (Time 1) to the clinic after discharge and at 3,6,12, and 18 months visit to the clinic after initiating TAM.

In this study, out of the 210 patients, we selected 135 patients who have prescribed TAM and were pre-menopausal. Those who do not meet the menopause criteria were classified as pre-menopause. Criteria for determining menopause included any of the following: age ≥ 60 years or age < 60 years with amenorrhea for ≥ 12 months in the absence of prior chemotherapy or receipt of TAM and Follicle-stimulating hormone (FSH) in the post-menopausal range (≥ 30 mIU/ml) (5). We classified data collected at 3 and 6 months as Time 2 and that at 18 and 24 months as Time 4. Therefore, Time 1 represents the data collected upon TAM initiation at baseline, and Time 2 represents data at 3–6 months, Time 3 at 12 months, and Time 4 at 18–24 months after initiating TAM. Finally, 104 patients without missing data were used in the analysis.

At Time 1, we collected patients’ general characteristics from electronic medical records with their consent, including age, educational level, employment status, prior history of cancer, family history of cancer, cancer stage, and treatment process (type of surgery, chemotherapy, and radiation therapy).

### Measures

HFS, SP, JMD, and PME symptoms were measured using the Korean version of the menopause rating scale (MRS) (22). The scale comprises three dimensions: somato-vegetative, psychological, and urogenital symptoms, with 11 items. Each item was scored using the following 5-point Likert scale: no symptom = 0, mild = 1, moderate = 2, severe = 3, and extremely severe = 4. A higher total score indicated greater self-reported menopausal symptoms. The scale’s Cronbach’s *α* was 0.88.

We assessed adherence to TAM. Patients were asked to rate their adherence rates from 0% to 100% from the last prescription date to the date of reporting medication adherence from Time 2 to Time 4. A 100% rating meant that the patient had taken medication every day, and a 0% rating meant that the patient had not taken any medication on any day.

### Statistical analysis

Statistical analyses were performed using SPSS software (version 26.0; SPSS Inc., Chicago, IL, USA). Participant characteristics were analyzed using descriptive analysis. Furthermore, we analyzed the proportion of patients reporting a score of =1 and ≥2 for each of the four symptoms and the number of patients according to the number of symptoms reporting a score of ≥1 and ≥2 over the four time points using descriptive analysis. Symptom changes on the four time points and interaction effects between participant characteristics and symptoms were analyzed using repeated measures analysis of variance (ANOVA), including the Bonferroni adjustment in the *post-hoc* analysis. Furthermore, the chi-square test was used to evaluate the differences between “no symptom to mild” and “moderate-to-extremely severe” at Time 2, according to the chemotherapy treatment experiences of patients who reported a “no symptom or mild” score at Time 1.

## Results

**Table 1** presents an overview of the participants’ characteristics. A total of 104 premenopausal patients diagnosed with BC and receiving TAM therapy were included in this study. The mean age was 43.5 ± 6.9 years, and 79.8% of the patients were aged > 40 years. Approximately 60% of them had a university education or above (64.4%), and 55.8% were employed. A high percentage of patients did not have prior (95.2%) or family (67.3%) history of cancer. Most patients were diagnosed with stage I, II, or III (81.7%), and 83.7% underwent conservation surgery. In the course of their treatment, 32.7% and 86.5% of patients received chemotherapy and radiation therapy, respectively. The overall patients’ TAM adherence was highest at Time 2 with an average of 96.2% (median of 100%) and the lowest at Time 3 with an average of 91.9% (median of 99%).

**Table 1:** Overview of participant characteristics.

Characteristics		n (%) or Mean (SD)
Age (years)		43.5 (6.9)
	< 40	21 (20.0)
	≥ 40	83 (79.8)
Education level	High school or below	37 (35.6)
	University and above	67 (64.4)
Employment status	Unemployed	46 (44.2)
	Employed	58 (55.8)
Prior history of cancer	No	99 (95.2)
	Yes	5 (4.8)
Family history of cancer	No	70 (67.3)
	Yes	34 (32.7)
Cancer stage	In situ	19 (18.3)
	Invasive (Stage I, II, or III)	85 (81.7)
Type of surgery	Mastectomy	17 (16.3)
	Conservation	87 (83.7)
Chemotherapy	Not done	70 (67.3)
	Done	34 (32.7)
Radiation therapy	Not done	14 (13.5)
	Done	90 (86.5)
Adherence to TAM (%)	Time 2	96.2 (14.1)
	Time 3	91.9 (21.8)
	Time 4	95.0 (11.6)

**Figure 1:**
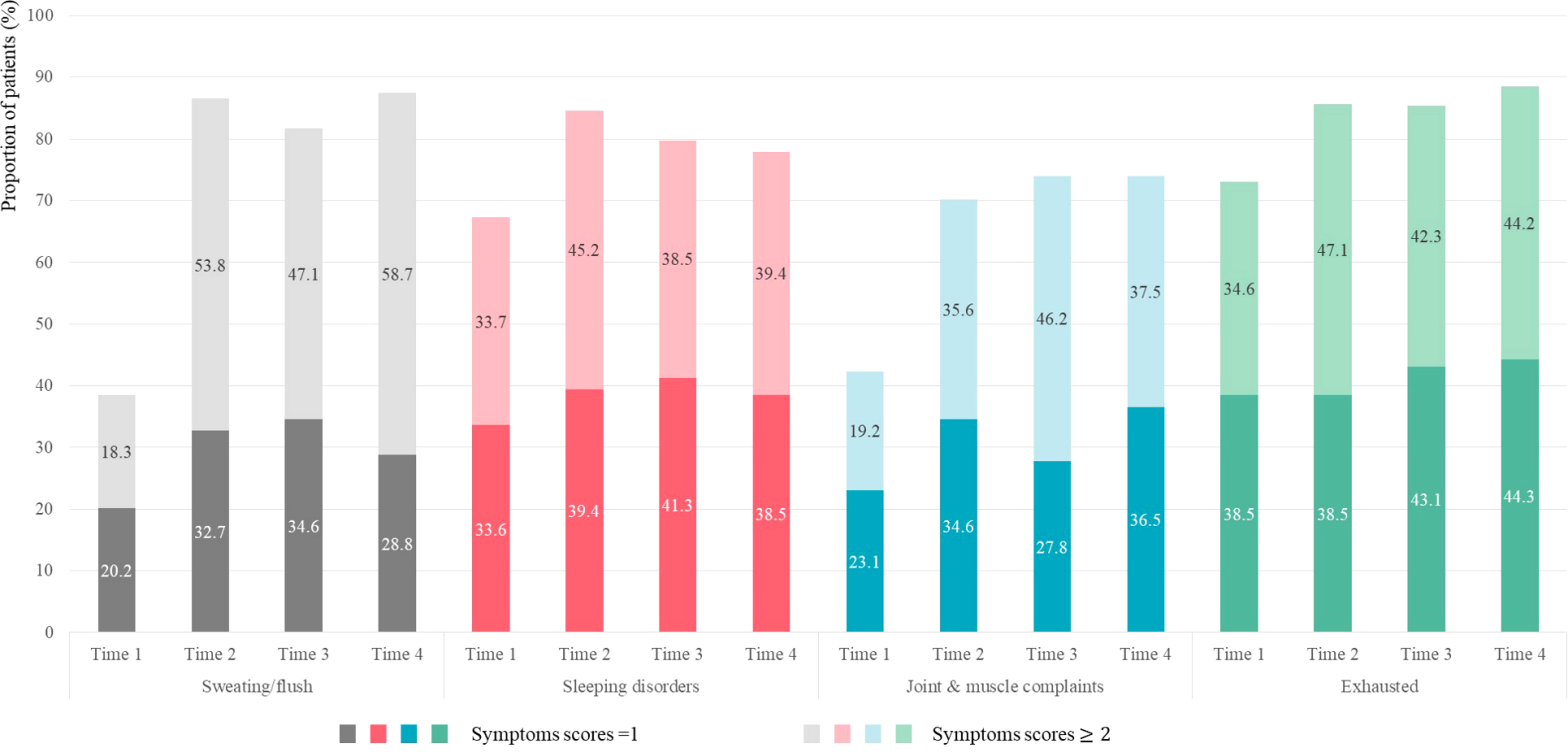
Proportion of patients reporting “mild” and “moderate-to-extremely severe” scores for HFS, SP, JMD, and PME symptoms.

**Figure 1** describes the proportion of patients reporting “mild” and “moderate-to-extremely severe” MRS scores for HFS, SP, JMD, and PME symptoms over four time points. All four symptoms were reported in > 70% patients after initiating TAM from Time 2 to Time 4. HFS exhibited the greatest increase, from 38.5% at Time 1 to 86.5% at Time 2. The proportion of patients with “moderate-to-extremely severe” MRS scores increased from 53.8% at Time 2 to 58.7% at Time 4. SP and PME demonstrated a high proportion from 67.3% and 73.1% at Time 1 compared to the other two symptoms.

**Figure 2:**
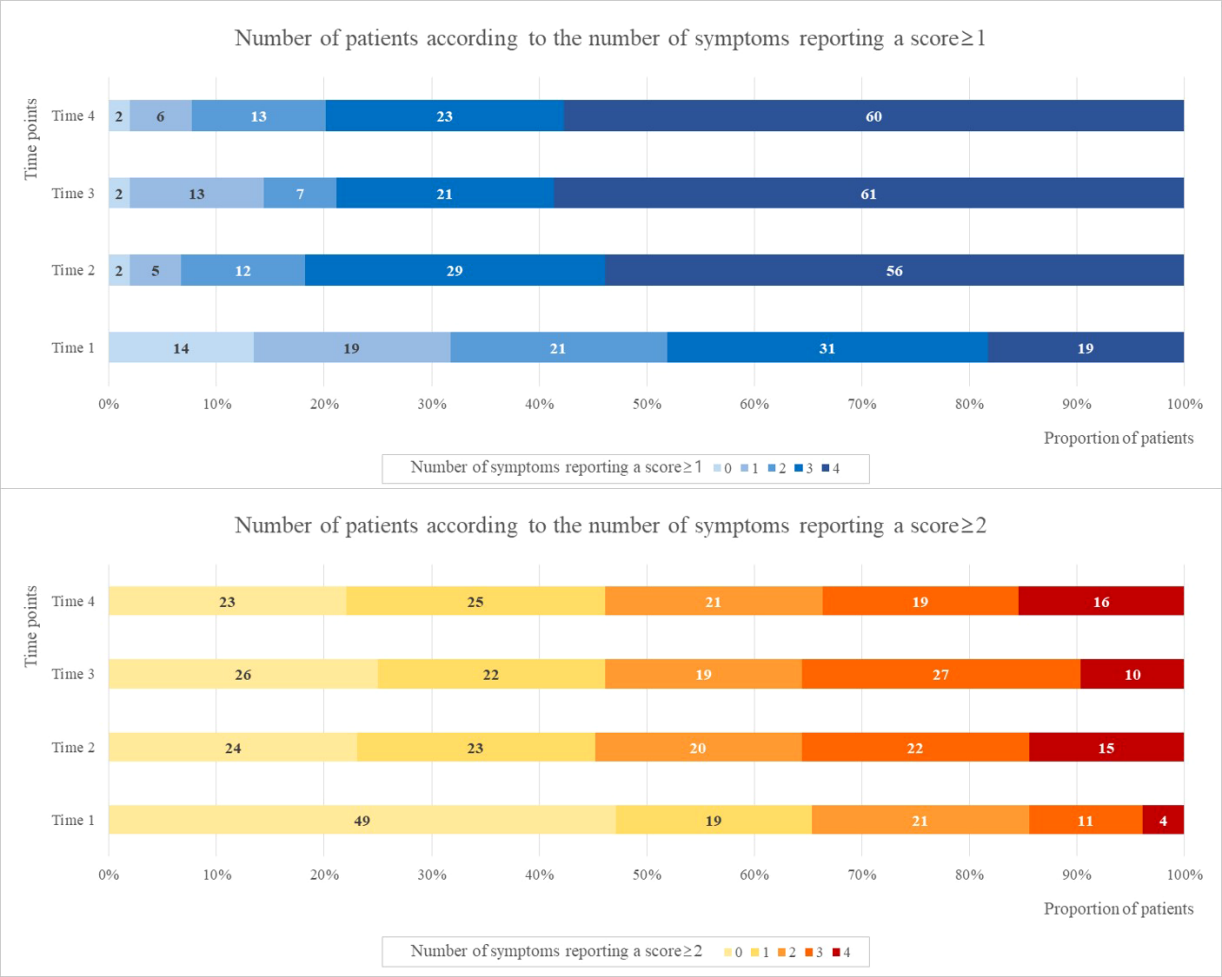
The number of patients according to the number of symptoms reporting a score ≥1 and ≥2 at four time points.

**Figure 2** presents the number of patients according to the number of symptoms reporting “mild-to-extremely severe” (≥1) and “moderate-to-extremely severe” (≥2) scores over the four time points. The number of patients who scored ≥ 1 for all four symptoms were 19 (18.3%), 56 (53.8%), 61 (58.7%), and 60 (57.7%) at Times 1, 2, 3, and 4, respectively. The number of patients who scored ≥1 for all four symptoms at Time 1 increased approximately 3 times at Time 2 and remained constant until Time 4. Approximately 55% of patients taking TAM experienced four mild-to-extremely severe symptoms simultaneously after taking TAM.

The number of patients with scores ≥ 2 for two or more symptoms were 36 (34.6%), 57 (54.8%), 56 (53.9%), and 56 (53.9%) at Times 1, 2, 3, and 4, respectively. The number of patients with scores ≥ 2 for at least two symptoms at Time 1 increased approximately 1.5 times at Time 2 and remained constant until Time 4. Approximately 50% of patients taking TAM experienced two or more symptoms after taking TAM.

**Table 2** presents the repeated measures ANOVA results with a Greenhouse-Geisser correction for the four menopausal symptoms at the four time points. There were significant changes in all symptoms: HFS (*p*<.001), SP (*p*<.001), JMD (*p*<.001), and PME (*p=*.004). Since initiating TAM, HFS exhibited the highest mean scores. SP showed the highest at Time 2, JMD at Time 3, and PME at Time 4. *Post hoc* analysis with a Bonferroni adjustment revealed that HFS and JMD were statistically significantly increased from Time 1 to Time 2 (*p*<.001), 3 (*p*<.001), and 4 (*p*<.001). SP were statistically significantly increased from Time 1 to Time 2 (*p=*.004) and PME were statistically significantly increased from Time 1 to Time 2 (*p=*.026) and 4 (*p=*.023).

**Table 2:** Symptom scores at four time points.

Menopausal symptoms	Time 1	Time 2	Time 3	Time 4	F	*p*
Hot flushes and sweating	0.64	1.71	1.52	1.77	47.731	<.001
Sleep problems	1.17	1.55	1.39	1.40	4.435	<.001
Joint and muscular discomfort	0.72	1.27	1.44	1.31	13.824	<.001
Physical and mental exhaustion	1.18	1.48	1.38	1.51	4.711	.004

**Table 3** presents the interaction effects of participant characteristics on patient-reported symptoms at the four time points. There were statistically significant interaction effects between age and time on SP change (*p*=.019). Patients aged < 40 years demonstrated significantly higher SP scores than those aged > 40 years after initiating TAM therapy. There were significant interaction effects between chemotherapy and time on JMD change (*p*=.025). Patients who had undergone chemotherapy exhibited significantly higher JMD scores than those who had not over the four time points.

**Table 3:** Interaction effects of participant characteristics on patient-reported menopausal symptoms over four time points.

Symptoms	Characteristics		Time 1	Time 2	Time 3	Time 4	F	*p*
Sleep problems	Age	< 40	1.05	1.90	1.57	1.90	3.496	.019
		≥ 40	1.20	1.46	1.35	1.28		
Joint and muscular discomfort	Chemotherapy	Done	1.06	1.76	1.56	1.32	3.169	.025
		Not done	0.56	1.03	1.39	1.30		

**Table 4** presents the differences in menopausal-symptom scores at Time 2 according to the chemotherapy treatment experiences of patients who reported “no symptom-to-mild” at Time 1. The results revealed no significant differences between “no symptom-to-mild” and “moderate-to-extremely severe” at Time 2 according to patients’ chemotherapy-treatment experiences. However, among patients who had undergone chemotherapy, 56.5% and 43.5% who either did not or mildly experienced HFS and JMD at Time 1 experienced “moderate-to-extremely severe” symptoms at Time 2, respectively.

**Table 4:** Menopausal-symptom scores at Time 2 according to chemotherapy in patients who reported “no symptom-to-mild” scores at Time 1.

Symptoms	Chemotherapy	MRS scores in Time 2		χ^2^	*p*
		0–1	2–4		
Hot flushes and sweating	Done	10 (43.5)	13 (56.5)	1.006	.316
	Not done	34 (55.7)	27 (44.3)		
Sleep problems	Done	15 (78.9)	4 (21.1)	1.090	.296
	Not done	33 (66.0)	17 (34.0)		
Joint and muscular discomfort	Done	13 (56.5)	10 (43.5)	3.449	.063
	Not done	47 (77.0)	14 (23.0)		
Physical and mental exhaustion	Done	10 (66.7)	5 (33.3)	0.326	.568
	Not done	31 (58.5)	22 (41.5)		

Data are presented as number of patients (%).

## Discussion

This study’s findings provide 24-month trajectories of four menopausal symptoms, including HFS, SP, JMD, and PME, experienced by premenopausal women diagnosed with BC taking TAM in Korea. This study further provides longitudinal symptom trends of BC in Korean women, with BC incidence being higher in the early 40s and > 50% of patients being premenopausal women, whereas in western countries, BC incidence is predominantly in the 50s (11).

In this study, patient adherence to TAM was found to be relatively high (> 91%), which is consistent with a previous study that reported > 94% of Korean patients with BC (21), although up to 50% of patients with BC are generally known not to take TAM for the full duration (23). A previous study has suggested that this result is due to the ceiling effect. In this study, as TAM adherence was high, menopausal symptoms, which were side effects of TAM, were also prevalent in many patients from 3 months to 24 months after initiating TAM administration. All four menopausal symptoms were reported in > 70% of patients with BC after initiating TAM and persisted until 24 months. More than 50% of patients experienced four menopausal symptoms, with at least two at a serious severity level 3–6 months after initiating TAM.

HFS occurred in > 80% of patients after initiating TAM, consistent with previous studies in which TAM-prescribed patients with BC patients had a prevalence of approximately 80% (10, 24). We found that the proportion of patients and mean scores exhibited the greatest increase at 3–6 months after initiating TAM. These increases did not continue beyond 24 months; however, they seemed consistent up to 24 months after taking TAM, indicating that HFS persists for a long time. Among the four menopausal symptoms, HFS symptom intensity was found to be predominant. We also found that > 47% of patients experienced moderate-to-extremely severe symptoms after taking TAM, and this proportion increased to > 55% at 18–24 months after initiating TAM, a figure that was slightly lower than the 60% reported in previous studies on patients with BC patients taking TAM (10, 25). This paper adds the following new information: among patients who have no or mild HFS before commencing TAM, > 55% of those who had undergone chemotherapy deteriorated to “moderate-to-extremely severe” at 3–6 months after initiating TAM. This result may provide evidence that corroborates previous research in which HFS and chemotherapy were not associated with postmenopausal women but possibly with premenopausal women (10). HFS has been found to be the greatest factor for intentional non-adherence to AET as well as negatively affect the quality of life of women with BC (25, 26). When prescribing TAM in clinical practice, it is necessary to consider the possibility of HFS occurrence in most patients, and that > 50% of these patients experience severe symptoms, especially in those who would have undergone chemotherapy.

JMD occurred in > 70% of patients after taking TAM, consistent with previous studies on TAM-prescribed patients with BC that indicated a prevalence of approximately 70% (20). We found a significant interaction effect between chemotherapy and time on JMD change. Patients who underwent chemotherapy exhibited higher JMD scores from baseline than those who did not undergo chemotherapy, and they experienced more amplified JMD after commencing TAM treatment. Even among patients with no or mild JMD, > 40% of those who underwent chemotherapy have been shown to experience a worsening of symptoms 3–6 months after initiating TAM, a figure that is approximately twice that of those who have never undergone chemotherapy. This study’s results emerged due to the fact that various types of chemotherapy cause JMD (27–29), and the symptoms apparently worsened as TAM therapy was initiated. JMD is known to have a significant impact on patient quality of life (30, 31), suggesting that active medical intervention is required.

Interestingly, SP and PME occurred in > 60% patients from baseline and persisted until 24 months after TAM. These two symptoms have been known to worsen with the initiation of chemotherapy or radiation therapy and were cumulative over the treatment course (32–34). Previous studies have already demonstrated that SP and PME are correlated (19, 35, 36). Thus, the reason underlying the high proportions and mean scores of SP and PME from baseline seems to be the patients’ treatment history, such as previous chemotherapy, radiation therapy, or surgery, among others.

Regarding SP, we found interaction effects between age and time on change. Patients aged < 40 years were found to experience greater SP than those aged > 40 years. Our results conflict with those of previous studies, showing solid evidence that sleep is more fragmented as we age, and that increasing age is associated with poorer sleep (37, 38). The reason this conflict exists is probably that younger women are more likely to present advanced disease and/or undergo aggressive treatment regimens (39), both of which potentially lead to severe SP.

This study has limitations. First, patients in this study were recruited from one tertiary hospital in Seoul, Korea, limiting the generalizability of the findings. Second, sample bias is possible because missing data were excluded. Third, medication TAM adherence and the four menopausal symptoms were assessed using patients’ self-reports, which are not exempt from self-presentation or memory bias, thus leading to inaccurate estimations of actual adherence and symptom changes (40). Fourth, although healthcare providers provided further interventions such as drug therapy, or consultation with proper specialists according to the symptom severity complained of by the patients with BC taking AET, these interventions were not considered in the analysis of this study.

## Conclusion

The present study investigated 24-month trajectories of four menopausal symptoms experienced by premenopausal women diagnosed with BC taking TAM in Korea. After initiating TAM therapy, all menopausal symptoms occurred in > 70% of BC patients. Patient symptoms increased at 3–6 months and persisted until 24 months after initiating TAM. More than 50% of patients experienced four menopausal symptoms, with at least two at a serious severity level. HFS occurred in the highest number of patients with high scores. SP and PME demonstrated relatively high scores, even before initiating TAM therapy. Young patients aged < 40 years experienced more severe SP than those aged > 40 years. Patients who had previously undergone chemotherapy experienced JME at a more severe and faster rate than those who had never undergone chemotherapy over the four time points. The present findings may assist in alerting healthcare providers to menopausal symptoms during TAM therapy and the need for early and active intervention to minimize symptom occurrence and distress. Appropriate interventions to manage SP in young women aged < 40 years and JMD in those who would have undergone chemotherapy should be implemented when initiating TAM. In particular, in the case of HFS and JMD, even if patients who have already undergone chemotherapy experience no symptoms before initiating TAM, severe symptoms may appear after TAM is initiated; therefore, active intervention is necessary. Furthermore, healthcare providers should be aware that patients may have high underlying SP and PMEs even before initiating TAM.

## Data availability statement

The original contributions presented in the study are included in the article/supplementary material. Further inquiries can be directed to the corresponding author.

## Ethics statement

The studies involving human participants were reviewed and approved by Institutional Review Board of Asan Medical Center (2016-0351 and 2018-1249). The patients/participants provided their written informed consent to participate in this study.

## Author contributions

Conceptualization: SS, YHM, SKP, and SBL; Methodology: SS, YHM, and SKP; Formal analysis and investigation: SS, YHM, and SKP; Writing—original draft preparation: SS; Writing—review and editing: SS, YHM, SKP, and SBL; Funding acquisition: YHM; Final approval of manuscript: SS, YHM, SKP, and SBL. All authors contributed to the article and approved the submitted version.

## Funding

This work was supported by the National Research Foundation (NRF) of Korea grant funded by the Korean government (Grant number NRF-2018R1A1A3A04076879).

## Conflict of interest

The authors declare that the research was conducted in the absence of any commercial or financial relationships that could be construed as a potential conflict of interest.

## Publisher’s note

All claims expressed in this article are solely those of the authors and do not necessarily represent those of their affiliated organizations, or those of the publisher, the editors and the reviewers. Any product that may be evaluated in this article, or claim that may be made by its manufacturer, is not guaranteed or endorsed by the publisher.
